# Supplementary material for: The Weight of Migration: Reconsidering Health Selection and Return Migration among Mexicans
Source: Int J Environ Res Public Health. 2021 Nov 19;18(22):12136. doi: 10.3390/ijerph182212136 (PMC8624630; doi:10.3390/ijerph182212136)
Supplement: Supplementary file 1 [file ijerph-18-12136-s001.zip › ijerph-1429348-supplementary.pdf]

International Journal of
Environmental Research and Public Health

MDPI

Table S1. Mixed Effects Model of the Association between Cardiometabolic Health and Future Migration to the USA, MxFLS Wave 1, n = 14,763.

	**Model 1**			**Model 2**			**Model 3**			**Model 4**			**Model 5**			**Model 6**		
**Future Migrant to the US Migrant = 1; Nonmigrant = 0**	**Elevated Waist Circumference**			**Elevated Mean Arterial Pressure**			**Diabetes**			**Cardiovascular Disease**			**Smoker**			**Physical Activity**		
	**OR**	**s.e.**	**95% CI**	**OR**	**s.e.**	**95% CI**	**OR**	**s.e.**	**95% CI**	**OR**	**s.e.**	**95% CI**	**OR**	**s.e.**	**95% CI**	**OR**	**s.e.**	**95% CI**
Age	0.91†	0.01	0.89 − 0.93	0.91†	0.01	0.89 − 0.93	0.91†	0.01	0.89 − 0.93	0.91†	0.01	0.89 − 0.93	0.91†	0.01	0.89 − 0.93	0.91†	0.01	0.89 − 0.93
Female	0.49†	0.10	0.33 − 0.73	0.43†	0.09	0.29 − 0.64	0.45†	0.09	0.31 − 0.67	0.45†	0.09	0.30 − 0.66	0.47†	0.10	0.32 − 0.70	0.46†	0.09	0.31 − 0.69
Married	0.66†	0.14	0.44 − 0.99	0.64†	0.13	0.42 − 0.96	0.64†	0.13	0.43 − 0.97	0.65†	0.13	0.43 − 0.97	0.64†	0.13	0.43 − 0.97	0.65†	0.14	0.43 − 0.98
Education (None = Ref)																		
Primary School	1.23	0.31	0.74 − 2.02	1.25	0.32	0.76 − 2.06	1.24	0.32	0.75 − 2.04	1.24	0.32	0.76 − 2.05	1.24	0.32	0.75 − 2.04	1.24	0.31	0.75 − 2.04
Secondary School	1.18	0.32	0.69 − 1.99	1.21	0.32	0.71 − 2.04	1.21	0.32	0.72 − 2.05	1.22	0.33	0.72 − 2.05	1.21	0.33	0.72 − 2.05	1.19	0.32	0.70 − 2.01
Currently Working	0.93	0.19	0.62 − 1.39	0.93	0.19	0.62 − 1.40	0.94	0.19	0.63 − 1.40	0.94	0.19	0.63 − 1.40	0.93	0.19	0.62 − 1.39	0.94	0.19	0.63 − 1.40
Health Insurance	0.42†	0.10	0.27 − 0.66	0.42†	0.10	0.26 − 0.65†	0.42	0.10	0.27 − 0.66	0.42†	0.10	0.27 − 0.66	0.42†	0.10	0.27 − 0.66	0.42†	0.10	0.27 − 0.66
Assets (Owns Home = Ref)	1.22	0.36	0.69 − 2.17	1.22	0.36	0.68 − 2.16	1.22	0.35	0.69 − 2.15	1.21	0.35	0.69 − 2.14	1.24	0.36	0.70 − 2.20	1.20	0.35	0.68 − 2.13
Urban	0.48*	0.18	0.23 − 1.01	0.48*	0.18	0.22 − 1.01	0.48*	0.18	0.23 − 1.01	0.48*	0.18	0.23 − 1.01	0.47*	0.18	0.22 − 1.00	0.47*	0.18	0.22 − 1.00
Return Migrant	5.88†	2.27	2.76 − 12.53	5.78†	2.23	2.71 − 12.30	5.72†	2.20	2.69 − 12.16	5.62†	2.16	2.64 − 11.95	5.73†	2.21	2.69 − 12.18	5.68†	2.18	2.67 − 12.07
Elevated Waist Circumf.	0.61*	0.16	0.37 − 1.02	--	--	--	--	--	--	--	--	--	--	--	--	--	--	--
Elevated MAP	--	--	--	0.78	0.15	0.53 − 1.15	--	--	--	--	--	--	--	--	--	--	--	--
Diabetes	--	--	--	--	--	--	1.05	0.55	0.38 − 2.95	--	--	--	--	--	--	--	--	--
Cardiovascular Disease	--	--	--	--	--	--	--	--	--	2.20	1.20	0.76 − 6.38	--	--	--	--	--	--
Smoker	--	--	--	--	--	--	--	--	--	--	--	--	1.35	0.36	0.81 − 2.27	--	--	--
Physical Activity	--	--	--	--	--	--	--	--	--	--	--	--	--	--	--	1.34	0.33	0.84 − 2.16

Mixed effects logistic regression models predicting future migration to the US. Model 1 − 6 each health indicator entered alone as an independent variable. † Indicates significance at $p < 0.05$ level, * indicates $p < 0.10$. Model adjusted for level 1 clustering at the family level, and level 2 clustering at the locality level.

Table S2. Mixed Effects Model of the Association between Time in the US and Waist Circumference, MxFLS Wave 1, n = 14,763.

DV: Waist Circumference	b	s.e.	95% CI	
Age	0.21†	0.01	0.19	0.22
Female	− 3.98†	0.21	− 4.39	− 3.57
Married	4.51†	0.20	4.11	4.90
Education (None = Ref)				
Primary School	− 0.15	0.26	− 0.66	0.37
Secondary School + †	− 1.94†	0.28	− 2.49	− 1.40
Currently Working	0.95†	0.21	0.53	1.36
Health Insurance	0.67†	0.21	0.25	1.08
Assets (Owns Home = Ref)	0.32	0.28	− 0.23	0.87
Urban	0.71	0.61	− 0.49	1.90
Smoker	− 0.90†	0.27	− 1.44	− 0.36
Physical Activity	− 1.17†	0.27	− 1.70	− 0.64
Time in the US (ref = 0 years)				
0.16 − 1.16yrs	− 0.58	1.32	− 3.17	2.01
1.25 − 2yrs	2.33*	1.31	− 0.24	4.90
2.16 − 5 years	3.39†	1.36	0.72	6.06
5.75 + years	2.39*	1.48	− 0.51	5.29

† Indicates significance at $p < 0.05$ level, * indicates $p < 0.10$; Models are adjusted for level 1 clustering at the family level and level 2 clustering at the locality level.
